# Immunohistochemical Analysis of Brainstem Lesions in the Autopsy Cases with Severe Motor and Intellectual Disabilities Showing Sudden Unexplained Death

**DOI:** 10.3389/fneur.2016.00093

**Published:** 2016-06-27

**Authors:** Masaharu Hayashi, Hiroshi Sakuma

**Affiliations:** ^1^Department of Brain Development and Neural Regeneration, Tokyo Metropolitan Institute of Medical Science, Tokyo, Japan

**Keywords:** severe motor and intellectual disabilities, sudden unexplained death, brainstem, immunohistochemistry, catecholamine, substance P, c-fos

## Abstract

It is known that patients with severe motor and intellectual disabilities (SMID) showed sudden unexplained death (SUD), in which autopsy failed to identify causes of death. Although the involvement of brainstem dysfunction is speculated, the detailed neuropathological analysis still remains to be performed. In order to clarify pathogenesis, we investigated the brainstem functions in autopsy cases of SMID showing SUD. We immunohistochemically examined expressions of tyrosine hydroxylase, tryptophan hydroxylase, substance P, methionine-enkephalin, and c-fos in the serial sections of the midbrain, pons, and medulla oblongata in eight SUD cases and seven controls, having neither unexplained death nor pathological changes in the brain. Expressions of tyrosine hydroxylase and tryptophan hydroxylase were reduced in two of eight cases, and those of substance P and/or methionine-enkephalin were augmented in the pons and medulla oblongata in seven of eight cases, including the aforementioned two cases, when compared with those in controls. The hypoglossal nucleus and/or the dorsal vagal nucleus demonstrated increased neuronal immunoreactivity for c-fos in seven of eight cases, although there was no neuronal loss or gliosis in both the nuclei. Controls rarely showed immunoreactivity for c-fos in the medulla oblongata. These data suggest the possible involvement of brainstem dysfunction in SUD in patients with SMID, and consecutive neurophysiological evaluation of brainstem functions, such as all-night polysomnography and blink reflex, may be useful for the prevention of SUD, because some parameters in the neurophysiological examination are known to be related to the brainstem catecholamine neurons and the spinal tract nucleus of trigeminal nerve.

## Introduction

Severe motor and intellectual disabilities (SMID) describe a heterogeneous group of disorders with severe physical disabilities and profound mental retardation (1, 2). Patients with SMID are frequently complicated with various neurological disorders and respiratory problems, such as upper airway obstruction, aspiration, and central apnea (3–5), in addition to circulatory disturbances. In patients with SMID, it is known that sudden unexplained death (SUD) accounts for 5% of cause of death (6), and definite causes are not identified even after autopsy in some cases. The involvement of brainstem dysfunction is speculated in sudden infant death syndrome (SIDS) (7–9), and disturbances of brainstem catecholamine neurons were involved in sudden death in developmental brain disorders, such as SIDS and Fukuyama-type congenital muscular dystrophy (FCMD) (10, 11). The brainstem expression of substance P was augmented in the spinal tract nucleus of trigeminal nerve in SIDS victims and cases of FCMD showing SUD (10, 12). In addition, the altered expression of proto-oncogene c-fos, a marker of early neuronal activation subsequent to noxious stimulation, was observed in sudden death in neurological disorders (12–14). Herein, we performed the comprehensive immunohistochemistry on the brainstem lesions in autopsy cases, in order to clarify the pathogenesis of SUD in patients with SMID, and discussed the possibility of monitoring SUD using neurophysiological examination.

## Patients and Methods

The clinical subjects comprised eight cases of SMID showing SUD (the SUD cases) between 1968 and 2001 at Tokyo Metropolitan Fuchu Medical Center for the Disabled and Tokyo Metropolitan Neurological Hospital and seven controls, aged from 7 to 55 years, showing neither SUD nor pathological changes in the central nervous system (Table 1). The family of each subject provided informed consent to the detailed neuropathological analysis. The brains were fixed in a buffered formalin solution, and each formalin-fixed brain was cut coronary, embedded in paraffin. We examined the cerebral cortex and white matters, basal ganglia, thalamus, cerebellum, brainstem, and spinal cord by routine histochemical staining and failed to find neither neuronal loss nor gliosis in each region. Considering the possibility of functional lesions in the brainstem, which have been pointed out in SIDS and/or FCMD, we performed immunohistochemistry in the brainstem sections subsequently. For immunohistochemical staining of the brainstem, sections with thickness of 5 μm were serially cut in the upper and lower parts of the midbrain, and the upper, middle, and lower parts of the pons, and medulla oblongata. They were deparaffinized, quenched with 1% hydrogen peroxide, and treated after microwave antigen retrieval with the following antibodies: mouse monoclonal antibodies to glial fibrillary acidic protein (GFAP, Dako, Glostrup, Denmark), tyrosine hydroxylase (Affinity Bioreagents, Inc., Golden, CO, USA), and tryptophan hydroxylase (Oncogen Research Product, Cambridge, MA, USA), in addition to rabbit polyclonal antibodies to substance P (Zymed Laboratories, Foster City, CA, USA), methionine-enkephalin (Chemikon International, Inc., Temecula, CA, USA), and c-fos (Santa Cruz Biotechnology, CA, USA) at the following concentrations: 1:50 (GFAP, substance P), 1:100 (tryptophan hydroxylase), 1:400 (tyrosine hydroxylase), 1:1000 (methionine-enkephalin, c-fos). Antibody binding was visualized by means of the avidin–biotin–immunoperoxidase complex method (Nichirei, Tokyo, Japan) following the manufacturer’s protocol. No staining was confirmed in sections in the absence of either antibody. In immunohistochemistry for tyrosine hydroxylase and tryptophan hydroxylase, counts of immunoreactive neurons were performed by the manual-labeling of appropriate cells with nuclei in two serial sections, and the mean value were calculated in each brainstem region (Table 2). The mean and SD of averages in the controls and SUD cases was analyzed with *t*-test for the comparison, and reported *p* values were shown in Table 2. This study was approved by the Ethical Committee in Tokyo Metropolitan Institute of Medical Science.

**Table 1:** **Summary of clinical and pathological findings**.

No.	Age (year)/sex	Brain disorders	Cause of death	Resuscitation time (h)	Postmortem time (h)	Brain weight (g)	Increase of goblet cells
**Controls**							
1	7/male	(−)	Sepsis		nd	nd	(−)
2	9/male	(−)	Sepsis		nd	nd	(−)
3	10/male	(−)	Leukemia		4	nd	(−)
4	22/male	(−)	DIC		nd	nd	(−)
5	29/male	(−)	Heart failure		nd	nd	(−)
6	38/male	(−)	Leukemia		nd	nd	(−)
7	55/male	(−)	Pulmonary cancer		5	1400	(−)
**Sudden unexplained death**							
1	6/male	Kernicterus		2	3	1331	1+
2	9/male	Schizencephaly		10	2	499	1+
3	13/male	Perinatal HIE		3	2	635	1+
4	20/male	Perinatal HIE		0.5	3	580	1+
5	23/female	Micro syndrome		1	7.5	260	(−)
6	29/female	Kernicterus		0.5	2	1437	1+
7	36/male	Postnatal HIE		2	6	578	1+
8	47/male	Encephalopathy		3	4	1348	(−)

*The degree of goblet cell change was graded by visual inspection as absence (−) or presence 1+. DIC, disseminated intravascular coagulation; HIE, hypoxic ischemic encephalopathy; nd, not determined*.

**Table 2:** **Summary of immunohistochemistry for tyrosine hydroxylase and tryptophan hydroxylase**.

No.	Tyrosine hydroxylase					Tryptophan hydroxylase	
	Periaqueductal gray matter	Locus ceruleus		Dorsal vagal nucleus		Superior central nucleus	Medullary raphe nucleus
		Left	Right	Left	Right		
**Controls**							
1	28	124.5	124.5	17.5	17	nd	34.5
2	19	88.5	91	6.5	7	170.5	49.5
3	10	nd	nd	10	12.5	nd	35.5
4	13.5	144.5	132.5	11	13.5	165	37
5	35	124	151	10.5	11.5	52	31
6	26.5	147	127	12	10	94.5	12.5
7	23.5	116.5	51	9	9.5	141	20.5
mean ± SD	22 ± 9	124 ± 21	113 ± 36	11 ± 3	12 ± 3	125 ± 50	32 ± 12
**Sudden unexplained death**							
1	34	94	88.5	11.5	13	164	59
2	7.5	**23**	**24.5**	**0**	**0**	110	28
3	28	136.5	136.5	7	8	80	13
4	35.5	98	114	10	13	67.5	56.5
5	29	137.5	138	12	14.5	119	13
6	5.5	**17**	**15.5**	**0**	**0**	53.5	**4.5**
7	25.5	76	73.5	7.5	11	55	47.5
8	17	85	79.5	19.5	18	93	16
mean ± SD	23 ± 12	83 ± 45	84 ± 46	8 ± 6	10 ± 7	93 ± 37	30 ± 22
*p* value (*t*-test)	0.92	0.06	0.23	0.38	0.51	0.22	0.85

*The number of tyrosine hydroxylase- and tryptophan hydroxylase-immunoreactive neurons in the each brainstem region is expressed. The mean of averages in controls and the SUD cases was analyzed with t-test for the comparison, and reported p values were shown in Table 2. Hatched labeling with bold characters denotes a reduction in the number of neurons less than the mean minus 2SD of average in controls*.

*nd, not determined*.

## Results

**Figure 1:**
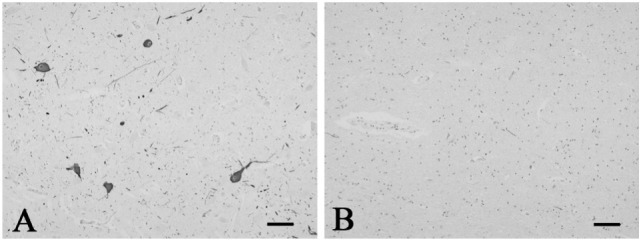
**Representative photographs in immunohistochemistry for tyrosine hydroxylase**. Immunoreactivity for tyrosine hydroxylase was observed in the neurons and neuronal processes in the periaqueductal gray matter in the control 5 **(A)**. The SUD case 6 showed a reduction in neurons and processes immunoreactive for tyrosine hydroxylase in the same area **(B)**. Bars = 80 μm.

**Figure 2:**
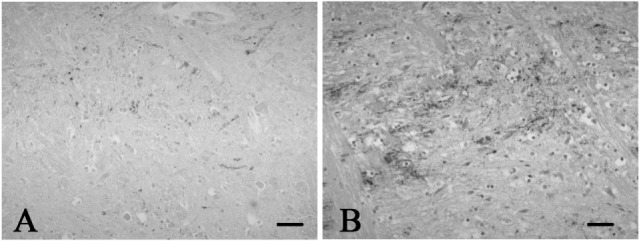
**Representative photographs in immunohistochemistry for substance P**. Immunoreactivity for substance P was observed in a few neuronal processes in the spinal tract nucleus of trigeminal nerve in the control 1 **(A)**, while that was found in many neuronal processes in the SUD case 2 **(B)**. Bars = 40 μm.

**Figure 3:**
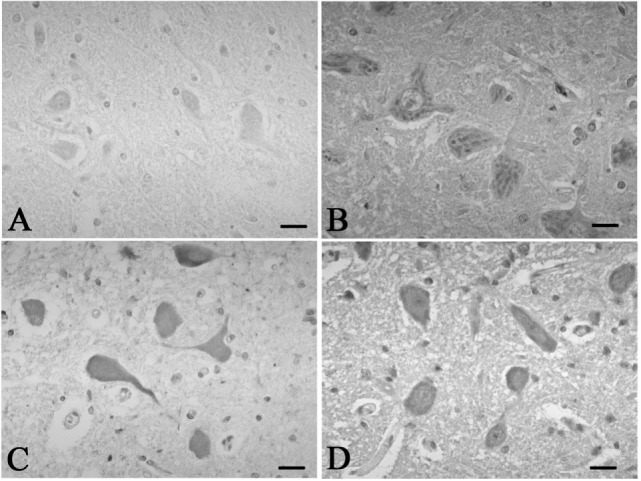
**Representative photographs in immunohistochemistry for c-Fos**. In the hypoglossal nucleus, there were no neurons immunoreactive for c-fos in the control 5 **(A)**, which was found in the SUD cases 5 **(B)**. Neurons immunoreactive for c-fos were observed in the dorsal vagal nucleus in both the control 4 **(C)** and SUD case 4 **(D)**. Bars = 20 μm.

A mild increase of the goblet cells was found in the SUD cases (Table 1), but that may be related to recurrent subtle aspiration, and did not lead to the diagnosis of bronchopneumonia. Neither neuronal loss nor increase of astrocytes immunoreactive for GFAP was observed in the brainstem regions examined in the SUD cases, in comparison with sections in controls. Neurons and neuronal processes immunoreactive for tyrosine hydroxylase were observed in the periaqueductal gray matter in the midbrain, locus ceruleus, and dorsal vagal nucleus in controls (Table 2; Figure 1A). Neurons immunoreactive for tryptophan hydroxylase were found in the superior central nucleus and raphe nucleus in the medulla oblongata in controls (Table 2). There was no significant difference in the number of neurons immunoreactive for either tyrosine hydroxylase or tryptophan hydroxylase between controls and the SUD cases. However, the SUD cases 2 and 6 exhibited a reduction in the number of neurons immunoreactive for tyrosine and/or tryptophan hydroxylase less than the mean minus 2SD of average in the controls (Table 2; Figure 1B). There was a variation in the degree of immunoreactivity for substance P and methionine-enkephalin in the neuropil and neuronal fibers in the substantia nigra in both controls and the SUD cases. Immunoreactivity for substance P was augmented in seven of eight SUD cases in the spinal tract nucleus of trigeminal nerve, in comparison with that in controls (Table 3; Figure 2). Four of the aforementioned seven SUD cases showed the increased immunoreactivity for substance P in the dorsal vagal nucleus. Moreover, the four of seven SUD cases with the augmented immunoreactivity for substance P also demonstrated increased immunoreactivity for methionine-enkephalin in the spinal tract nucleus of trigeminal nerve (Table 3). There were a few neurons immunoreactive for c-fos in the periaqueductal gray matter, pontine tegmentum, and posterior funicular nucleus in both controls and the SUD cases (Table 4). In the hypoglossal nucleus, neurons immunoreactive for c-fos were found in six of eight SUD cases, but not in controls (Figures 3A,B). In addition, five of the aforementioned SUD cases and the SUD case 1 showed neurons immunoreactive for c-fos in the dorsal vagal nucleus, in which those were observed only in the control 4 (Figures 3C,D).

**Table 3:** **Summary of immunohistochemistry for substance P and methionine-enkephalin**.

No.	Substance P			Methionine-enkephalin		
	Substantia nigra	Dorsal vagal nucleus	Spinal tract nucleus of trigeminal nerve	Substantia nigra	Dorsal vagal nucleus	Spinal tract nucleus of trigeminal nerve
**Controls**						
1	2+	1+	1+	1+	(−)	(−)
2	nd	1+	1+	nd	1+	(−)
3	2+	1+	1+	1+	1+	(−)
4	1+	1+	1+	1+	1+	1+
5	2+	1+	1+	2+	1+	1+
6	1+	1+	1+	1+	1+	1+
7	2+	1+	1+	2+	1+	1+
**Sudden unexplained death**						
1	2+	1+	1+	2+	1+	(−)
2	2+	1+	**2+**	1+	1+	(−)
3	2+	**2+**	**2+**	1+	1+	1+
4	1+	**2+**	**2+**	1+	1+	1+
5	2+	**2+**	**2+**	2+	1+	**2+**
6	2+	1+	**2+**	1+	1+	**2+**
7	1+	1+	**2+**	1+	1+	**2+**
8	2+	**2+**	**2+**	2+	1+	**2+**

*The degree of immunoreactivity of neuronal processes was graded as follows: absence as (−); a few as 1+; and many as 2+. Hatched labeling with bold characters denotes an augmentation of immunoreactivity compared with that in controls*.

*nd, not determined*.

**Table 4:** **Summary of immunohistochemistry for c-fos**.

No.	Periaqueductal gray matter	Pontine tegmentum	Posterior funicilar nucleus	Hypoglossal nucleus	Dorsal vagal nucleus
**Controls**					
1	1+	(−)	(−)	(−)	(−)
2	(−)	1+	(−)	(−)	(−)
3	1+	1+	(−)	(−)	(−)
4	1+	(−)	1+	(−)	1+
5	(−)	(−)	(−)	(−)	(−)
6	1+	1+	(−)	(−)	(−)
7	1+	1+	(−)	(−)	(−)
**Sudden unexplained death**					
1	1+	(−)	(−)	(−)	1+
2	(−)	(−)	(−)	(−)	(−)
3	1+	(−)	(−)	1+	1+
4	(−)	(−)	(−)	1+	1+
5	(−)	(−)	(−)	1+	1+
6	(−)	(−)	(−)	1+	(−)
7	(−)	(−)	1+	1+	1+
8	1+	(−)	(−)	1+	1+

*The absence and presence of neurons immunoreactive for c-fos are denoted as (−) and 1+*.

## Discussion

In the SIDS victims, fine morphological changes have been noted in the brainstem respiratory network, including Kölliker–Fuse nucleus (7–9), although autopsy failed to reveal coarse lesions in the brain. Similarly, the disturbances in the autonomic nervous system, fluctuating muscle tone with involuntary movements and/or hypersensitivity to environmental stimuli, may be involved in SUD in patients with neurological disorders (12), but the detailed pathogenesis still remains to be investigated. We previously reported the selective disturbance in the brainstem expressions of tyrosine hydroxylase, tryptophan hydroxylase, and methionine-enkephalin in autopsy cases of West and Lennox–Gastaut syndromes (15). In this analysis, the expressions of neurotransmitters, substance P, methionine-enkephalin, and c-fos were examined in serial brainstem sections in the SUD cases, focusing on the brainstem dysfunction. The SUD cases 2 and 6 seemed to show disturbances in catecholamine and serotonin neurons (Table 2). In addition, seven of the SUD cases including the cases 2 and 6 showed increased immunoreactivity for substance P and/or methionine-enkephalin in the spinal tract nucleus of trigeminal nerve (Table 3). Seven of the eight SUD cases demonstrated neurons immunoreactive for c-fos in the hypoglossal nucleus and/or dorsal vagal nucleus (Table 4). These immunohistochemical abnormalities lacked both obvious neuronal loss and gliosis, suggesting the possible disturbances of brainstem function.

Sudden infant death syndrome victims were reported to show reduced immunoreactivity for tyrosine hydroxylase in the dorsal vagal nucleus and ventrolateral reticular formation in the medulla oblongata (10). Sudden death sometimes occurs in patients with FCMD, in which the neurons immunoreactive for tyrosine hydroxylase were reduced in the reticular formation of medulla oblongata, dorsal vagal nucleus, and solitary tract nucleus (11, 12). Tyrosine hydroxylase is a rate-limiting enzyme in catecholamine biosynthesis, and the reduced expression of tyrosine hydroxylase in the brainstem are assumed to reflect the dysfunction of catecholamine neuron system, which can lead to disturbed autonomic regulation and/or neural respiratory control. The similar pathological processes can be speculated in the SUD cases 2 and 6 in this analysis. The medullary serotonin neurons, which are also involved in the autonomic and respiratory homeostasis, showed increased cell density with reduced binding receptor sites in SIDS victims (16). The SUD case 6 only showed a reduction of medullary serotonin neurons, and the lesion of serotonin neurons in the SUD cases seems to be less predominant and different from that in SIDS. Increased immunoreactivity for substance P associated with proliferation of astrocytes was found in the pontine reticular formation and the medullary spinal trigeminal nucleus in the SIDS victims (10). Inasmuch as substance P increases central inspiratory activity and can mediate the central nervous response to hypoxia, it is speculated that the augmented expression of substance P in the SIDS victims may be related to the influence of hypoxia. The augmented immunoreactivity for substance P was also observed in the spinal tract nucleus of trigeminal nerve and dorsal vagal nucleus in the SUD cases, but astrocytes immunoreactive for GFAP were not increased. In our previous study, the immunoreactivity for methionine-enkephalin, but not substance P, was reduced in the absence of neuronal loss and gliosis in the spinal tract nucleus of trigeminal nerve in the autopsy cases of West and Lennox–Gastaut syndromes (15). Although the exact mechanism of difference in the disturbed expressions of substance P and methionine-enkephalin between intractable epilepsy and SUD is not clear, it is suggested that the impairments of trigeminal system can be involved. C-fos is the first gene activated by noxious signals, particularly in the presence of hypoxia, and useful to demonstrate the stimulus-related local neuronal activation (13, 14). Neurons immunoreactive for c-fos were increased in the dorsal vagal nucleus in SIDS victims (13), and those were observed in the hypoglossal nucleus and/or the dorsal vagal nucleus in the SUD cases in this analysis. Since the hypoglossal nucleus and dorsal vagal nucleus are known to be associated with neuronal cardiopulmonary regulation (17), the increase of neurons immunoreactive for c-fos in SIDS victims and the SUD cases may reflect a certain response to hypoxic brain insults.

It is controversial whether the immunohistochemical changes in this analysis are causes or results of SUD; however, the data suggest the possible involvement of brainstem dysfunction in SUD. Patients with SMID occasionally demonstrate abnormalities in both the sleep parameters in all-night polysomnography and the R2 components in blink reflex, which are related to the brainstem catecholamine neurons and the spinal tract nucleus of trigeminal nerve, respectively, both of which demonstrated changes in the SUD cases of SMID in this analysis (18). We believe that routine neurophysiological evaluation of brainstem functions, including all-night polysomnography and blink reflex, will give us a clue to prevent SUD in patients with SMID. Furthermore, we will try to quantify the protein expressions by Western blot analysis in the frozen specimen of autopsy brains in the future.

## Conclusion

Sudden unexplained death remains to be one of the important causes of death in patients with SMID, although the exact pathomechanisms are not clarified fully. The data in this analysis suggest the possible involvement of brainstem dysfunction in SUD, and the detailed neurophysiological evaluation of brainstem functions is recommended in patients with SMID.

## Author Contributions

Prof. MH planned this analysis and conducted all processes, such as selection of subjects, immunohistochemical staining, quantitative assessment of each specimen, taking photographs, and writing the manuscript. Prof. HS financially supported this analysis, supervised all processes, and discussed the context of manuscript.

## Conflict of Interest Statement

The authors declare that the research was conducted in the absence of any commercial or financial relationships that could be construed as a potential conflict of interest.
